# A pre-post test evaluation of the impact of the PELICAN MDT-TME Development Programme on the working lives of colorectal cancer team members

**DOI:** 10.1186/1472-6963-10-187

**Published:** 2010-06-29

**Authors:** Cath Taylor, Joanna M Sippitt, Gary Collins, Chris McManus, Alison Richardson, Jeremy Dawson, Michael Richards, Amanda J Ramirez

**Affiliations:** 1 Florence Nightingale School of Nursing and Midwifery, King's College London, London, SE1 8WA, UK; 2 Promoting Early Presentation Group, Institute of Psychiatry, King's College London, Adamson Centre, St Thomas' Hospital, London, SE1 7EH, UK; 3 Centre for Statistics in Medicine, Wolfson College Annexe, University of Oxford, Oxford, OX2 6UD, UK; 4 Department of Psychology, University College London, Gower Street, London, WC1E 6BT, UK; 5 School of Health Sciences, University of Southampton and Southampton University Hospital Trust, Southampton, SO16 6YD, UK; 6 Work & Organisational Psychology Group, Aston Business School, Aston University Aston Triangle, Birmingham, B4 7ET, UK; 7 National Cancer Action Team, St Thomas' Hospital, London, SE1 7EH, UK

## Abstract

**Background:**

The PELICAN Multidisciplinary Team Total Mesorectal Excision (MDT-TME) Development Programme aimed to improve clinical outcomes for rectal cancer by educating colorectal cancer teams in precision surgery and related aspects of multidisciplinary care. The Programme reached almost all colorectal cancer teams across England. We took the opportunity to assess the impact of participating in this novel team-based Development Programme on the working lives of colorectal cancer team members.

**Methods:**

The impact of participating in the programme on team members' self-reported job stress, job satisfaction and team performance was assessed in a pre-post course study. 333/568 (59%) team members, from the 75 multidisciplinary teams who attended the final year of the Programme, completed questionnaires pre-course, and 6-8 weeks post-course.

**Results:**

Across all team members, the main sources of job satisfaction related to working in multidisciplinary teams; whilst feeling overloaded was the main source of job stress. Surgeons and clinical nurse specialists reported higher levels of job satisfaction than team members who do not provide direct patient care, whilst MDT coordinators reported the lowest levels of job satisfaction and job stress. Both job stress and satisfaction decreased after participating in the Programme for all team members. There was a small improvement in team performance.

**Conclusions:**

Participation in the Development Programme had a mixed impact on the working lives of team members in the immediate aftermath of attending. The decrease in team members' job stress may reflect the improved knowledge and skills conferred by the Programme. The decrease in job satisfaction may be the consequence of being unable to apply these skills immediately in clinical practice because of a lack of required infrastructure and/or equipment. In addition, whilst the Programme raised awareness of the challenges of teamworking, a greater focus on tackling these issues may have improved working lives further.

## Background

The National PELICAN Multidisciplinary Team Total Mesorectal Excision (MDT-TME) Development Programme was a unique team-based residential training course attended by colorectal multidisciplinary team members across England. The Programme originated as a series of workshops funded by Macmillan Cancer Support. It was designed to teach surgeons the technical skills to complete total mesorectal excision (TME) by live demonstration of the surgery using video conferencing facilities, and thereby aimed to improve the quality and usage of TME surgery. The success of these initial workshops led to the NHS commissioning a fully multidisciplinary programme for all members of colorectal multidisciplinary teams[1]. The demonstration of TME remained central to the Development Programme which also aimed to provide education about relevant aspects of radiology, histology, oncology and nursing.

We took the opportunity to evaluate the impact of participating in this national team-based course on the working lives of colorectal team members. Underpinning this study was the drive to tackle the high levels of poor mental health reported among UK cancer doctors. In 2002 the estimated prevalence of psychiatric morbidity (GHQ-12 scores ≥4) among UK hospital consultants was 33% compared with 27% in 1994, according to a national cohort study[2,3]. This deterioration in mental health over an eight year period was especially marked in clinical and surgical oncologists and explained by increased job stress without a comparable increase in job satisfaction. Similarly high levels of poor mental health have recently been reported amongst colorectal surgeons and nurse specialists in the UK NHS[4]. On the basis of findings that MDT working may be beneficial to the mental health and working lives of team members[5], we evaluated the impact of participating in this novel MDT-based Development Programme on levels of job stress, levels of job satisfaction and team performance of colorectal cancer team members.

## Methods

### Study design and participants

We conducted a pre-post course evaluation involving all core team members from the 75 teams attending one of the 11 courses in the final year of the programme (September 2005 to October 2006). Core team members included consultant surgeons, oncologists, histopathologists and radiologists, as well as clinical nurse specialists and MDT coordinators.

### Procedures

We ascertained team members through the PELICAN Cancer Foundation, the charity coordinating the Development Programme. Pre-course questionnaires were posted and e-mailed to each core team member approximately three weeks before each course, with a reminder two weeks later. Post-course questionnaires were sent four weeks after each course, with reminders sent six and eight weeks post-course. Consent to participate in the study was assumed by return of a completed questionnaire. Confidentiality was maintained by the allocation of a unique identification number, necessary for matching pre- and post-course responses. Approval for this study was granted by South East Research Ethics Committee and by the Trust Research and Development Department for each multidisciplinary team.

### The Development Programme

In total, 35 courses were held from June 2003 to October 2006. 183 out of 186 colorectal multidisciplinary teams in England attended (1639 delegates in total). Courses were held on a monthly basis and an average of seven teams attended each course.

Each course lasted two days, over which time team members received education about the management of rectal cancer from national leaders in the field. Central to the course was the opportunity to observe live TME surgery with two-way discussion between operating and lecture theatres using video conferencing facilities. There were also seminars on the latest advances in imaging[6,7], pathology[8,9], pre-operative radio and chemo-radiotherapy[10], supportive care issues including stoma management, and the management of secondary disease. One session was dedicated to the challenges of multidisciplinary team working. This session was unique to each course and chaired by members of the teams in attendance. Team members had the opportunity for social interaction throughout the course by virtue of its residential nature, with overnight accommodation for two nights, together with a course dinner.

### Outcomes

Change in self-reported job stress, job satisfaction, and team performance between pre- and post-course assessment.

#### Job stress and job satisfaction

We measured self-reported job stress and job satisfaction using an adapted version of The Hospital Consultants' Job Stress and Satisfaction Questionnaire[11]. The resulting study-specific questionnaire comprised items common to all multidisciplinary team members (46 items related to job stress and 44 items related to job satisfaction) and items relevant only to team members who provide direct patient care: surgeons, oncologists and clinical nurse specialists (10 job stress items and 10 job satisfaction items). See additional file 1 for job stress and satisfaction questionnaire.

Job stress and satisfaction item scores: Each questionnaire item was rated according to the extent it had contributed to an individual's total job stress or total job satisfaction on a scale from 0 (not at all) to 3 (a lot).

Job stress and satisfaction sources scores: Factor analysis was used to explore the grouping of individual stress and satisfaction items. All job satisfaction items aggregated to one of seven main sources of satisfaction. Most job stress items aggregated to one of eight main sources of job stress. Three job stress items did not aggregate to a main source of job stress: '*feeling poorly paid for the job you do'; 'being required to provide routine clinical NHS services outside of normal working hours' *and '*disruption to home-life due to on-call work'*. Job stress in relation to these items is included in the calculation of total job stress but is not reported individually. Scores for the main sources of job stress and job satisfaction were calculated by summing ratings given to the individual items that aggregated to each source and were standardised (on a scale of 0-100). For each score presented, the higher the score the higher the level of job stress or job satisfaction.

Total job stress and satisfaction scores: Total job stress and job satisfaction scores were calculated by summing ratings given to all job stress and job satisfaction items respectively. Scores were standardised (on a scale of 0-100) to aid comparison between team members who did and did not provide direct patient care.

#### Team performance

Key aspects of team performance were measured the Aston Team Performance Inventory[12] (ATPI). Each team member rated 33 statements on a five-point scale from 'Strongly Disagree' to 'Strongly Agree'. Total team performance scores were calculated by aggregating responses to all 33 statements. Individual statements also aggregated to one of six domains of team-working (table 1). Scores were standardised to enable comparison across domains. The ATPI is designed for use as a team-level measure and therefore for each team in the study, the response from individual members was averaged to give team-based total and domain scores.

**Table 1:** Impact of participation in the MDT-TME Development Programme on job stress, job satisfaction and team performance

	*Pre-course*	*Post-course*	*Estimated difference between pre- and * *post-course mean scores (95% confidence interval)*	*p*
			
	*Mean score*			
**Total job stress**	38	36	-2.4 (-3.5 to -1.2)	<*0.0001*
**Sources of job stress: *all team members***				
Feeling overloaded with work and impact on home-life	48	45	-2.5 (-4.3 to -0.8)	*0.01*
Service configuration and lack of resources	42	40	-1.8 (-3.2 to -0.4)	*0.01*
Being concerned about making clinical mistakes and its consequences	33	30	-2.7 (-4.6 to -0.9)	*0.004*
Dealing with changes to the way you do your job	32	31	-1.5 (-3.3 to 0.2)	0.08
Being unable to provide best quality care to patients	29	27	-1.6 (-3.3 to 0.1)	0.07
Having difficulties in relationships with people you work with	28	25	-2.9 (-4.7 to -1.0)	*0.003*
**Sources of job stress:** team members who provide direct patient care*				
Dealing with angry or blaming patients/relatives	48	40	-7.1 (-10.1 to -4.2)	<*0.0001*
Having distressed patients	36	33	-2.8 (-5.1 to -0.5)	*0.02*

**Total job satisfaction**	63	61	-2.7 (-4.1 to -1.4)	<*0.0001*
**Sources of job satisfaction: *all team members***				
Working in a multidisciplinary team	70	68	-1.4 (-3.3 to 0.6)	0.17
Providing better care from working in a multidisciplinary team	70	68	-2.2 (-4.4 to -0.1)	0.05
Having good relationships with colleagues beyond multidisciplinary team members	67	66	-1.5 (-3.4 to 0.5)	0.14
Nature of job	60	56	-3.4 (-4.9 to -2.0)	<*0.0001*
Aspects of the wider organisation	46	42	-3.5 (-5.3 to -1.7)	*0.0001*
**Sources of job satisfaction:** team members who provide direct patient care*				
Having good relationships with patients	84	81	-2.7 (-5.0 to -0.4)	*0.02*
Providing quality care to patients	77	74	-2.6 (-4.5 to -0.7)	*0.01*

	***Mean team score***			
**Total team performance**	70	71	1.6 (0.5 to 2.7)	*0.006*
**Aspects of team performance:**				
Task focus *(having a shared focus, constructive debate, shared aim* *for excellence)*	77	77	0.8 (-0.6 to 2.3)	0.26
Team conflict † *(disagreement, heated debates, interpersonal conflict)*	74	74	0.6 (-1.0 to 2.2)	0.46
Participation *(involvement of all team members, trusting supportive environment)*	72	73	0.7 (-0.5 to 1.9)	0.24
Creativity *(idea generating, trying out new things)*	69	71	1.6 (-0.2 to 3.3)	0.07
Managing *(how well the team leader organises and coordinates team,* *monitors quality, progress and provides feedback)*	68	69	0.8 (-0.7 to 2.4)	0.28
Reflexivity *(reviewing team's objectives, discussing how team is working)*	59	61	2.0 (0.3 to 3.7)	*0.02*

* completed by surgeons, oncologists and clinical nurse specialists only; † scores reversed (higher score = less conflict)

#### Pre-course expectations of the Development Programme and reported adequacy of skills training for effective multidisciplinary team working

At the pre-course assessment, team members completed an open-ended question: '*What are you hoping to gain from the PELICAN MDT-TME Development Programme?*' At that assessment team members also reported whether they felt adequately trained in communication skills, teamworking skills, handling complaints from patients and relatives, and team leadership.

### Statistical Methods

We assessed the impact of participation in the Development Programme on job stress, job satisfaction and team performance by analysing responses from team members who responded to both pre- and post-course questionnaires (n = 333). Analysis of team performance excluded one team as only one member responded to both pre- and post-course questionnaires.

We used repeated measures ANOVA to assess the impact of participation in the Development Programme on team members' job satisfaction and job stress (total levels and individual sources) and team performance. Due to the exploratory nature of this study no adjustment was made for multiple testing. Analysis was conducted using SPSS v.15 and R v.2.6.0.

Responses to the pre-course open-ended question about team members' expectations of the Development Programme were read and main themes described independently by four members of the research team. Once themes were agreed, one member of the research team coded each response according to the agreed framework.

## Results

### Participant flow

Between September 2005 and October 2006, 568 team members from 75 teams participated in the Development Programme at one of 11 courses. 464/568 (82%) team members responded to the pre-course questionnaire and 367/568 (65%) to the post-course questionnaire. A total of 333 team members (59%) responded to both pre- and post- course questionnaires. The average number of respondents per team was 7 (range 1 to 10).

### Characteristics of team members

Team members who completed both pre- and post- course questionnaires and those who were lost to follow-up had very similar baseline characteristics, including having similar levels of total job stress and job satisfaction (table 2). 104 team members participated in the Development Programme but did not complete either pre- or post- course questionnaires. Of these, 67 (65%) were male; 46 (45%) were surgeons and 21 (20%) were clinical nurses specialists.

**Table 2:** Demographic and professional characteristics of colorectal team members

	*Team members who completed pre- and post- course questionnaires* *n = 333 (%)*	Team members who completed pre-course questionnaire only n = 131(%)
**AGE GROUP**		
25 and under	2 (1)	1 (1)
26 - 35 years	35 (11)	14 (11)
36 - 45 years	159 (48)	48 (38)
46 - 55 years	102 (31)	48 (38)
Over 55 years	33 (10)	14 (11)
*Missing*	*2*	*6*
		
**GENDER**		
Male	176 (53)	74 (57)
Female	157 (47)	57 (44)
		
**MARITAL STATUS**		
Single	28 (8)	11 (9)
Married/cohabiting	285 (86)	104 (83)
Separated/divorced/widowed	18 (5)	10 (8)
*Missing*	*2*	*6*
		
**PROFESSIONAL GROUP**		
Surgeons	94 (28)	45 (34)
Oncologists	35 (11)	15 (12)
Radiologists	57 (17)	15 (12)
Histopathologists	27 (8)	14 (11)
Clinical nurse specialists	95 (29)	32 (24)
MDT coordinators	25 (8)	10 (8)
	***Mean score***	
**TOTAL JOB STRESS** **(pre-course)**	38	39
**TOTAL JOB SATISFACTION** **(pre-course)**	63	63

Over half of the team members who participated in the evaluation of the Programme were clinical nurse specialists or surgeons. Overall, the proportions of male and female team members were similar, but over 90% of MDT coordinators and clinical nurse specialists were female, whereas 90% of surgeons and 79% of radiologists were male.

Most surgeons and nurses were core members of only one multidisciplinary team, but the majority of oncologists, radiologists and histopathologists were core members of between two and four teams. Five percent of team members were core members of between five and nine different teams; over half of these were oncologists.

### Job stress and job satisfaction among colorectal cancer teams members at the pre-course assessment

Among all team members '*feeling overloaded with work and impact on home life' *was the most frequently reported source of job stress. '*Dealing with angry or blaming patients/relatives' *was associated with similarly high levels of job stress amongst team members who provide direct patient care (table 1). Across the professional groups surgeons reported the highest total job stress although only significantly higher than MDT coordinators. MDT coordinators reported the lowest job stress; significantly lower than all other professional groups (F_5,327 _= 4.9, p < 0.001). This pattern of differences in levels of total job stress across the professional groups was repeated for each of the individual sources of job stress (table 3).

**Table 3:** Impact of participation in the MDT-TME Development Programme on job stress and job satisfaction by professional group

	*Surgeon*		*Oncologist*		*Radiologist*		*Histo-pathologist*		*Clinical Nurse Specialist*		*MDT coordinator*		*Change between pre- and post-course scores according to professional group (p)*
		
	*pre*	*post*	*pre*	*post*	*pre*	*post*	*pre*	*post*	*pre*	*post*	*pre*	*post*	
		
	*Mean scores*												
**Total job stress**	40	39	38	36	40	37	39	39	37	34	25	23	0.50
**Sources of job stress:** ***all team members***													
Feeling overloaded with work and impact on home-life	51	50	46	42	53	49	52	53	44	40	34	32	0.58
Service configuration and lack of resources	45	44	44	40	45	41	46	46	38	37	32	29	0.76
Concern about making clinical mistakes and its consequences	34	33	22	24	41	37	49	45	31	28	14	9	0.52
Changes to the way you do your job	27	27	33	30	38	35	44	40	37	33	10	12	0.42
Being unable to provide best quality care to patients	41	38	43	42	21	17	10	9	27	25	7	7	0.83
Having difficulties in relationships with people you work with	27	27	26	25	29	23	23	24	31	25	25	21	0.15
**Sources of job stress:** ***team members who provide direct*** patient care*													
Dealing with angry or blaming patients/relatives	44	40	55	44	-	-	-	-	48	39	-	-	0.21
Having distressed patients	31	30	28	31	-	-	-	-	43	36	-	-	0.01^+^

**Total job satisfaction**	67	65	64	63	62	56	56	55	69	66	41	40	0.39
**Sources of job satisfaction:** ***all team members***													
Working in a multidisciplinary team	74	72	70	69	73	66	71	71	69	70	50	50	0.21
Providing better care from working in a multidisciplinary team	72	68	69	67	68	66	66	69	77	74	48	50	0.66
Having good relationships with colleagues beyond multidisciplinary team members	67	68	70	72	68	62	55	52	72	70	57	56	0.16
Nature of job	66	63	57	57	59	53	54	52	64	60	30	28	0.25
Aspects of the wider organisation	46	44	46	44	45	40	42	40	50	45	37	36	0.57
**Sources of job satisfaction:** ***team members who provide direct*** patient care*													
Having good relationships with patients	83	80	82	79	-	-	-	-	86	84	-	-	0.97
Providing quality care to patients	74	72	72	70	-	-	-	-	83	79	-	-	0.50

*completed by surgeons, oncologists and clinical nurse specialists only

+ estimated difference: CNS -7; 95% CI -11 to -3, p < 0.001; surgeons -1, 95% CI -5 to 2, p = 0.5; oncologists +3, 95% CI -3 to +9, p = 0.3

The highest levels of job satisfaction were reported by team members who provide direct patient care and related to '*having good relationships with patients' *and '*providing quality care to patients'*. Across all team members '*working in a multidisciplinary team' *and '*providing better care from working in a multidisciplinary team' *were the predominant sources of job satisfaction (table 1). Surgeons and clinical nurse specialists reported the highest job satisfaction; significantly higher than all other team members except oncologists. MDT coordinators reported the lowest satisfaction; significantly lower than all other team members (F_5,326 _= 16.5; p < 0.001). As with job stress, the pattern of job satisfaction levels by professional group for the individual sources of job satisfaction was similar to the pattern for total job satisfaction (table 3).

### Impact of the Development Programme on job stress, job satisfaction and team performance

Across all team members, levels of total job stress decreased after participation in the Development Programme (table 1). Levels of job stress decreased in six out of the eight individual sources (table 1). The decrease in total job stress was similar across all professional groups, as was the decrease in relation to seven of the eight individual source of job stress (table 3).

Levels of total job satisfaction also decreased after participation in the Development Programme (table 1). Levels of job satisfaction decreased in four out of the seven individual sources (table 1). The decrease in total job satisfaction and each individual source of job satisfaction was similar across each professional group (table 3).

There was a small increase in team performance scores after participation in the Development Programme. This was mostly accounted for by an increase in one aspect of team performance: the extent to which the team reflected on their objectives and the way their team was working (reflexivity; table 1).

### Team members' expectations regarding the Development Programme

278 (83%) team members responded to the pre-course question about their expectations of the Development Programme. The majority (240/278; 87%) gave a response that aggregated to one of two main themes:

• To assess or improve their team working (n = 206, 74%): to assess their team working in relation to others; or improve their team working (e.g. improve relationships within the team, increase efficiency, manage conflict or gain a better understanding of other team members' roles):

'Reassurance we are working together effectively and that our discussions are broadly in line with national thinking'

'How to make the MDT more effective, efficient and professional with more input from all members'

'*An increased awareness of the responsibilities of the other disciplines'*

'*I hope that as a result of the Programme our team may become more cohesive and be more functional, respectful and supportive'*

• To improve their knowledge (n = 80, 29%): to be educated about best practice, latest advancements, and training or learning specific to their role.

'*To improve my knowledge and skills in colorectal cancer management'*

'To improve my skills and understanding of MRI staging of rectal cancer and to improve the quality of the imaging'

### Adequacy of skills training for effective multidisciplinary teamworking

Prior to attending the Development Programme, three quarters of team members felt adequately trained in communication skills but this varied from 86% of nurses to only just over half of radiologists (table 4). Two-thirds of team members felt adequately trained in teamworking skills. Less than 40% of respondents felt adequately trained in dealing with complaints, with particularly low proportions of radiologists (19%) and MDT coordinators (16%) feeling adequately trained. Less than a third of team members felt adequately trained in team leadership.

**Table 4:** Adequacy of skills training for effective multidisciplinary teamworking

*Professional Group*	*Communication skills* *n (%)*	*Teamworking* *n (%)*	*Handling complaints* *n (%)*	Team leadership n (%)
**Surgeons**	76 (81)	64 (68)	49 (52)	43 (46)
**Oncologists**	27 (77)	18 (51)	15 (43)	13 (37)
**Radiologists**	31 (54)	33 (58)	11 (19)	11 (19)
**Histopathologists**	18 (67)	17 (63)	9 (33)	6 (22)
**Clinical nurse specialists**	82 (86)	68 (72)	42 (44)	23 (24)
**MDT coordinators**	18 (72)	15 (60)	4 (16)	2 (8)

**TOTAL (n = 333)**	**252 (76)**	**215 (65)**	**133 (39)**	**98 (29)**

## Discussion

For the colorectal team members we evaluated, participating in the MDT-TME Development Programme was associated with a decrease in average levels of job stress and job satisfaction six to eight weeks post-course. Team members also reported a marginal improvement in team performance. The magnitude of the decrease in team members' levels of job stress and job satisfaction was similar to the increase in job stress reported by UK hospital consultants between 1994 and 2002 using the same measures[3]. The increase in job stress explained a five percent increase in prevalence of estimated psychiatric morbidity among UK hospital consultants over that time period (from 27% to 32% with GHQ-12 scores ≥ 4). This suggests that the impact of the Development Programme on the well-being of colorectal team members is meaningful. The improvement in team performance scores was mainly explained by a change in the extent to which teams reflected on their teamworking. This has face validity given the nature of the Programme. Its importance is however difficult to interpret as the Aston Team Performance Inventory has yet to be validated against measures of the effectiveness of multidisciplinary teams including the mental health of team members.

These findings are challenging to interpret. The decrease in team members' job stress may reflect the improved knowledge and skills that the Development Programme conferred. The decrease in job satisfaction may be the consequence of being unable to apply these newly acquired skills in the immediate aftermath of the Development Programme because of a lack of required infrastructure and/or equipment. Early reports suggest that attendance at the Development Programme has improved clinical practice, including increased usage of MRI preoperatively (Brown, personal communication, 27.05.10); improved quality of reporting for radiology and increased lymph-node harvest (Yorkshire Audit Data, Quirke, personal communication, 08.06.10). An internal evaluation of the impact of the Development Programme on clinical practice 6-12 months after participation[13] also reports improved usage and reporting of MRI subsequent to attendance, as well as the adoption of new network-wide policies and protocols. However, some teams also reported being unable to implement some of the recommendations of the Programme due to workforce shortages. These included dual consultant operating for difficult cases and not having an MDT coordinator or clinical nurse specialist as part of the team.

Across all team members the main sources of job satisfaction related to working in a multidisciplinary team and providing better patient care as a result of multidisciplinary teamworking. This finding is consistent with findings from a national survey completed by over 2000 MDT members in England, of whom 90% agreed that MDT-working is beneficial to the mental health and wellbeing of team members, and 81% agreed that being an MDT member improves job satisfaction[14]. The perception that teamworking improves patient care fits with the emerging evidence that multidisciplinary teams are beneficial in terms of disease management and clinical outcomes[15].

The levels and nature of job stress and satisfaction among hospital consultants has already been well described[2,3] but this study provided a unique opportunity for comparison across all core colorectal members of multidisciplinary teams. We found that clinical nurse specialists reported similarly high levels of job stress as their medical colleagues. These high levels for nurses may reflect a lack of clarity about the content and boundaries of their job leading to excessive expectations from others[16-19]. They also reported high job satisfaction which is likely to be derived from spending most of their working time delivering direct patient care[16], as well as the positive impact on their professional status and esteem of being recognised as a core member of the multidisciplinary team in recent cancer policy. Having professional status and esteem has been shown to be a key source of satisfaction for hospital consultants[2,3]. MDT coordinators, who prepare and organise the multidisciplinary team meetings, have a relatively new and rapidly expanding role[20]. The importance of MDT coordinators for effective MDT working has been acknowledged[20,21]. However, their low job stress and low job satisfaction suggests the need to standardise and professionalise their work[22].

Strengths of this study include the response from almost 60% of team members from a third of all colorectal cancer teams in England to both pre- and post-course questionnaires. We received completed questionnaires from 82% of delegates pre-course and found no difference in total job stress and satisfaction scores between those who responded to follow-up and those who did not. We also used well validated job stress and satisfaction measures, designed specifically for cancer health professionals[11]. The Development Programme reached almost every colorectal cancer team in England and thus we chose a pre-post evaluation design as a randomised controlled trial was not possible. Our evaluation focused on only the final year of the programme, which was for pragmatic reasons. The relatively short timeframe of follow-up may have impacted on the results. Benefits may have dissipated in the long-term. Alternatively, gains in job satisfaction may have been reported in the longer term, once the challenges of instigating the necessary service changes had been overcome.

The Development Programme was not intended to provide training in teamworking. Nevertheless, a large proportion of team members attended the course with expectations that it would do so. This study highlights significant shortfalls in the training that multidisciplinary team members reported they had received in the skills that are key to effective teamworking, namely in communication, teamworking, handling complaints and leadership. Amongst these skills, effective leadership is arguably the most critical to the success of teamworking, supported by the recommendation for standardised leadership training by Lord Darzi[23]. To address the outstanding need for teamwork training identified by colorectal team members, the methods used to improve communication skills in cancer health professionals[24] could be integrated into courses such as the Development Programme.

## Conclusions

Participation in the Development Programme had a mixed impact on the working lives of team members in the immediate aftermath of attending. Although challenging to interpret, the decrease in team members' job stress may reflect the improved knowledge and skills conferred to many participants by the Programme. The decrease in job satisfaction may be the consequence of being unable to apply these skills immediately in clinical practice because of a lack of required infrastructure and/or equipment. In addition, whilst the Programme raised awareness of the challenges of teamworking, a greater focus on tackling these issues in combination with enhancing clinical skills and knowledge may go further to improve the working lives of cancer multidisciplinary team members.

## Abbreviations

ANOVA: Analysis of variance; ATPI: Aston Team Performance Inventory; MDT: Multidisciplinary team; NHS: National Health Service; TME: Total Mesorectal Excision.

## Competing interests

The authors declare that they have no competing interests.

## Authors' contributions

All named authors have agreed to the submission of this manuscript and participated in this study to a sufficient extent to be named as authors. AJR and MR conceived the idea for the study; CT was the study lead and wrote the first draft of the paper; JS was the study coordinator; GC provided statistical support; CMc, AR and JD advised on aspects of study design and interpretation of findings. All authors participated in the critical revision of the article and have read and approved the final manuscript.

## Pre-publication history

The pre-publication history for this paper can be accessed here:

http://www.biomedcentral.com/1472-6963/10/187/prepub

## Supplementary Material

Additional file 1 **Adapted version of the hospital consultants' job stress and job satisfaction questionnaire**. The questionnaire used to assess cancer team members' job stress and job satisfaction. Click here for file
