# Supplementary material for: A pre-post test evaluation of the impact of the PELICAN MDT-TME Development Programme on the working lives of colorectal cancer team members
Source: BMC Health Serv Res. 2010 Jun 29;10:187. doi: 10.1186/1472-6963-10-187 (PMC2914033; doi:10.1186/1472-6963-10-187)
Supplement: Additional file 1 — Adapted version of the hospital consultants' job stress and job satisfaction questionnaire. The questionnaire used to assess cancer team members' job stress and job satisfaction. [file 1472-6963-10-187-S1.PDF]

KING'S College LONDON

Adapted version of the Hospital Consultants' Job Stress & Satisfaction Questionnaire (HCJSSQ)

The manual for the HCJSSQ can be found at: http://www.iop.kcl.ac.uk/iopweb/blob/downloads/locator/l_370_HCJSSQ_Manual.pdf

This is an adapted version of the HCJSSQ relevant for all cancer team members.

© Kings College London Promoting Early Presentation Group 2010

Stressful aspects of your work

We are interested in sources of job stress and satisfaction for all members of your MDT. We have therefore compiled a detailed list of sources that apply to all of the different professional groups in your team to allow for comparison between groups. Some sources appear in the stress and satisfaction sections as they may contribute to both.

Please rate each source of stress according to the extent that it has ***contributed*** *to your overall stress in the past few months. Please score 'Not at all' if a source of job stress is not applicable to your job.*

		Extent that a source of stress has ***contributed*** to your overall job stress in **the past few months**			
		Not at all	A Little	Quite A bit	A Lot
1	Encountering difficulties in relationships with junior medical staff	☐	☐	☐	☐
2	Feeling you have insufficient input into the management of your unit or institution (e.g. service redesign)	☐	☐	☐	☐
3	Disruption of your home life through spending long hours at work	☐	☐	☐	☐
4	Having inadequate equipment to do your job properly	☐	☐	☐	☐
5	Keeping up to date with current clinical and research practices	☐	☐	☐	☐
6	Having to take on more managerial responsibilities	☐	☐	☐	☐
7	Encountering difficulties in relationships with colleagues	☐	☐	☐	☐
8	Feeling under pressure to meet deadlines	☐	☐	☐	☐
9	Being responsible for the quality of the work of other staff	☐	☐	☐	☐
10	Encountering difficulties in relationships with administrative staff (e.g. secretaries)	☐	☐	☐	☐
11	Having too great an overall volume of work	☐	☐	☐	☐

		Extent that a source of stress has ***contributed*** to your overall job stress in **the past few months**			
		Not at all	A Little	Quite A bit	A Lot
12	Feeling you are poorly paid for the job you do	☐	☐	☐	☐
13	Encountering difficulties in relationships with NHS managers	☐	☐	☐	☐
14	Having conflicting demands on your time (e.g. patient care/management/research/College)	☐	☐	☐	☐
15	Having inadequate staff to do your job properly	☐	☐	☐	☐
16	Dealing with the threat of litigation	☐	☐	☐	☐
17	Disruption of your home life as a result of taking work home	☐	☐	☐	☐
18	Feeling that your accumulated skills and expertise are not being put to their best use	☐	☐	☐	☐
19	Disruption of your home life as a result of being on call	☐	☐	☐	☐
20	Having a conflict of responsibilities (e.g. clinical vs. managerial; clinical vs. research)	☐	☐	☐	☐
21	Uncertainty over the future funding of your unit/institution	☐	☐	☐	☐
22	Being responsible for the welfare of other staff	☐	☐	☐	☐
23	Having performance targets which are unrealistic or unattainable (e.g. due to lack of resources, tight deadlines)	☐	☐	☐	☐
24	Having to comply with increasing bureaucratic and regulatory procedures	☐	☐	☐	☐
25	Feeling concerned about keeping your skills up to date	☐	☐	☐	☐
26	Providing patient care within multi-disciplinary teams	☐	☐	☐	☐
27	Feeling that you are losing generalist skills as your job becomes more specialised	☐	☐	☐	☐

		Extent that a source of stress has ***contributed*** to your overall job stress in **the past few months**			
		Not at all	A Little	Quite A bit	A Lot
28	Having difficulties recruiting high calibre staff	☐	☐	☐	☐
29	Having insufficient formalised time for teaching, training and research	☐	☐	☐	☐
30	Having inadequate administration systems (e.g. not having access to all relevant patient data)	☐	☐	☐	☐
31	Undergoing job planning and performance appraisal	☐	☐	☐	☐
32	Being required to provide routine NHS clinical services (e.g. outpatient clinics) outside normal working hours	☐	☐	☐	☐
33	Being unable to do the job as well as you would like	☐	☐	☐	☐
34	Being concerned about missing the diagnosis of cancer	☐	☐	☐	☐
35	Having to contribute to treatment decisions where mistakes have severe consequences	☐	☐	☐	☐
36	Being unable to cure patients	☐	☐	☐	☐
37	Being unable to treat patients quickly	☐	☐	☐	☐
38	Feeling responsible for treatment toxicity caused by treatment you prescribe	☐	☐	☐	☐
39	Feeling insufficiently supported for the emotional demands of your job	☐	☐	☐	☐
40	Being expected to undertake tasks that are inappropriate to your role/job	☐	☐	☐	☐
41	Having to work across several hospital sites	☐	☐	☐	☐
42	Conflict between other team members or colleagues	☐	☐	☐	☐
43	Feeling that clinical priorities are distorted by targets	☐	☐	☐	☐
44	Undergoing peer review	☐	☐	☐	☐

		Extent that a source of stress has ***contributed*** to your overall job stress in **the past few months**			
		Not at all	A Little	Quite A bit	A Lot
45	Being unable to provide the best treatment (e.g. treatment not approved by NICE, financial constraints)	☐	☐	☐	☐
46	Impact of the European Working Times Directive	☐	☐	☐	☐

Job stress relating to direct patient/relative contact
The following sources of stress relate to having direct patient or relative contact in your job. If this does not apply to your job please go straight to the subsequent section (job satisfaction).

		Extent that a source of stress has ***contributed*** to your overall job stress in **the past few months**			
		Not at all	A Little	Quite A bit	A Lot
1	Being involved with the physical suffering of patients	☐	☐	☐	☐
2	Having to deal with distressed, angry or blaming relatives	☐	☐	☐	☐
3	Being involved with the emotional distress of patients	☐	☐	☐	☐
4	Dealing with patients or relatives having expectations of care that cannot be met	☐	☐	☐	☐
5	Dealing with angry or blaming patients	☐	☐	☐	☐
6	Dealing with patient/relative complaints about care you have provided	☐	☐	☐	☐
7	Having to break bad news to patients and their relatives	☐	☐	☐	☐
8	Being unable to control patients' symptoms	☐	☐	☐	☐
9	Dealing with patients whose symptoms are benign, but who remain anxious about having cancer	☐	☐	☐	☐
10	Caring for patients who refuse treatment	☐	☐	☐	☐

Satisfying aspects of your work

*Please rate each source of satisfaction according to the extent that it has **contributed** to your overall satisfaction in the past few months. Please score 'Not at all' if a source of job satisfaction is not applicable to your job.*

		Extent that a source of satisfaction has ***contributed*** to your overall job satisfaction in **the past few months**			
		Not at all	A little	Quite a bit	A Lot
1	Having a high level of responsibility	☐	☐	☐	☐
2	Being perceived to do the job well by your colleagues	☐	☐	☐	☐
3	Being able to bring about positive change in your unit/institution	☐	☐	☐	☐
4	Feeling there are the staff necessary to do a good job	☐	☐	☐	☐
5	Deriving intellectual stimulation from research	☐	☐	☐	☐
6	Having a high level of autonomy	☐	☐	☐	☐
7	Having opportunities for personal learning (e.g. developing clinical/research/management skills)	☐	☐	☐	☐
8	Having good relationships with other staff members	☐	☐	☐	☐
9	Having variety in your job	☐	☐	☐	☐
10	Feeling you have adequate financial resources to do a good job	☐	☐	☐	☐
11	Being involved in activities that contribute to the development of your profession	☐	☐	☐	☐
12	Feeling you have a high level of job security	☐	☐	☐	☐
13	Deriving intellectual stimulation from teaching	☐	☐	☐	☐

		Extent that a source of satisfaction has ***contributed*** to your overall job satisfaction in **<u>the past few months</u>**			
		Not at all	A little	Quite a bit	A Lot
14	Feeling you have adequate equipment to do a good job	☐	☐	☐	☐
15	Feeling your clinical experience is used to the full in the job you do	☐	☐	☐	☐
16	Feeling you are able to deliver the best available care	☐	☐	☐	☐
17	Feeling that your expertise is valued in your multi-disciplinary team	☐	☐	☐	☐
18	Being able to treat patients quickly	☐	☐	☐	☐
19	Knowing that clinical decisions have the support of multi-disciplinary team colleagues	☐	☐	☐	☐
20	Feeling that you are a good role model for junior staff	☐	☐	☐	☐
21	Feeling that your multi-disciplinary team meeting is well-organised and coordinated	☐	☐	☐	☐
22	Other team members or colleagues getting on with each other	☐	☐	☐	☐
23	Having a good relationship with your multi-disciplinary team members	☐	☐	☐	☐
24	Having a good relationship with staff/colleagues outside of your multi-disciplinary team/s	☐	☐	☐	☐
25	Having a good relationship with allied health professionals (e.g. social workers, occupational therapists)	☐	☐	☐	☐
26	Having a good relationship with administration staff	☐	☐	☐	☐
27	Having a good relationship with junior medical staff	☐	☐	☐	☐
28	Having a good relationship with consultant colleagues	☐	☐	☐	☐

		Extent that a source of satisfaction has ***contributed*** to your overall job satisfaction in **the past few months**			
		Not at all	A little	Quite a bit	A Lot
29	Having a good relationship with NHS managers	☐	☐	☐	☐
30	Having a good relationship with nurses	☐	☐	☐	☐
31	Having a good relationship with radiographers	☐	☐	☐	☐
32	Being able to learn from each other within multi-disciplinary teams	☐	☐	☐	☐
33	Having your opinion actively sought in your MDM	☐	☐	☐	☐
34	Feeling supported by other multi-disciplinary team members	☐	☐	☐	☐
35	Feeling that patient care is improved through multi-disciplinary team working	☐	☐	☐	☐
36	Feeling that clinical decision making is improved through multi-disciplinary team working	☐	☐	☐	☐
37	Helping patients through curing cancer	☐	☐	☐	☐
38	Being an expert in a specialist area	☐	☐	☐	☐
39	Having the opportunity to practice medicine privately	☐	☐	☐	☐
40	Providing patient care within multi-disciplinary teams	☐	☐	☐	☐
41	Being able to complete a difficult clinical procedure successfully	☐	☐	☐	☐
42	Feeling your contribution is valued by other multi-disciplinary team members	☐	☐	☐	☐
43	Feeling you have control over your daily working life	☐	☐	☐	☐
44	Feeling appreciated by management/your Trust for the work that you do	☐	☐	☐	☐

Job satisfaction relating to direct patient/relative contact
The following sources of satisfaction relate to having direct patient or relative contact in your job. If this does not apply to your job please go straight to the subsequent section.

		Extent that a source of satisfaction has ***contributed*** to your overall job satisfaction in **the past few months**			
		Not at all	A little	Quite a bit	A Lot
1	Having good relationships with patients	☐	☐	☐	☐
2	Making a difference to patients' quality of life	☐	☐	☐	☐
3	Feeling you deal well with relatives	☐	☐	☐	☐
4	Being perceived to do the job well by patients	☐	☐	☐	☐
5	Knowing about your patients' wellbeing after discharge from your care (e.g. into community or hospice care)	☐	☐	☐	☐
6	Receiving expressions of appreciation from patients or relatives	☐	☐	☐	☐
7	Being perceived to do the job well by relatives	☐	☐	☐	☐
8	Helping patients through controlling their symptoms	☐	☐	☐	☐
9	Feeling you manage death and dying well for patients	☐	☐	☐	☐
10	Being able to provide continuity of care to patients	☐	☐	☐	☐
